# Supplementary material for: Investigation of Effects of Novel Bifidobacterium longum ssp. longum on Gastrointestinal Microbiota and Blood Serum Parameters in a Conventional Mouse Model
Source: Microorganisms. 2024 Apr 22;12(4):840. doi: 10.3390/microorganisms12040840 (PMC11052112; doi:10.3390/microorganisms12040840)
Supplement: Supplementary file 1 [file microorganisms-12-00840-s001.zip › microorganisms-2927684-supplementary.pdf]

Table S1. Relative abundance (%) of faecal microbiota genera of mice during the 15- and 30-day intervention period

**Phylum**	**Genus**	**CG1**		**CG2**			**TG1**		**TG2**		
		**At start**	**On Day 15**	**At start**	**On Day15**	**On Day 30**	**At start**	**On Day 15**	**At start**	**On Day15**	**On Day 30**
Bacteroidota	*Muribaculaceae*	33.9	34.7	4.4	6.9	34.0	49.4	6.9	6.0	5.9	37.2
	Alistipes	19.6	1.0	18.5	12.3	13.0	13.4	20.7	11.0	9.8	0.9
	Prevotellaceae_UCG-1	2.0	1.0	0.0	1.2	1.8	1.8	4.7	0.0	2.9	1.4
	Bacteroides	0.0	6.4	1.8	8.9	7.3	9.9	9.0	15.0	13.5	0.6
	Muribaculum	0.0	0.0	0.0	0.0	0.0	0.5	0.0	0.3	0.0	0.0
Bacillota	*Bacillus*	4.0	0.0	0.0	0.0	0.7	1.4	0.0	0.9	0.0	0.0
	Lachnospiraceae A2	3.4	2.3	5.6	5.5	2.4	0.0	0.0	3.4	0.0	1.2
	Lactobacillus	2.9	0.6	1.2	1.2	0.6	1.4	1.8	0.7	1.2	1.2
	RF39	2.6	0.9	0.0	0.0	0.0	0.0	2.4	0.0	0.0	1.0
	*Lachnospiraceae_NK4A136*_group	2.3	23.9	32.1	24.1	1.1	2.8	2.2	29.3	25.7	22.9
	Oscillibacter	1.7	1.0	0.0	1.3	0.9	1.3	1.2	1.7	1.5	1.1
	*Clostridiales _vadin BB6*_group	1.4	1.2	1.9	0.9	1.0	0.0	1.7	0.0	1.4	0.8
	Lachnospiraceae_UCG-1	1.1	1.4	1.6	1.7	0.9	2.0	2.1	0.0	0.0	0.7
	Colidextribacter	1.1	1.0	1.3	0.9	0.8	0.0	1.0	1.1	1.3	0.7
	Streptococcus	0.7	0.0	0.0	0.0	0.0	0.0	0.0	0.0	0.0	0.0
	Clostridia_UCG-14	0.0	3.7	4.4	2.3	4.4	2.8	4.1	2.8	1.1	0.0
	Roseburia	0.0	3.4	3.5	2.7	0.0	0.6	3.2	0.9	7.9	5.6
	Marvinbryantia	0.0	0.5	0.0	0.0	0.0	0.0	0.0	0.0	0.0	0.0
	Staphylococcus	0.0	0.0	0.9	0.0	0.0	0.0	2.4	0.0	0.0	0.0
	*[Eubacterium]_xylanophilum*_group	0.0	0.0	0.7	0.7	0.3	0.0	0.0	0.5	0.6	1.0
	Lachnospiraceae_UCG-6	0.0	0.0	0.0	0.0	0.0	0.5	0.0	0.0	0.5	1.1
Actinomycetota	*Kocuria*	0.9	0.0	0.0	0.0	0.0	0.0	0.0	0.0	0.0	0.0
	Micrococcus	0.7	0.0	0.0	0.0	0.0	0.0	0.0	0.0	0.0	0.0
	Adlercreutzia	0.0	0.5	0.0	4.4	4.7	0.7	0.7	6.2	0.7	3.7
	Bifidobacterium	0.0	0.0	0.0	0.0	0.0	0.0	0.6	0.0	0.0	0.0
Candidatus Patescibacteria	*Candidatus Saccharimonas*	2.9	7.8	2.3	12.7	15.7	0.6	17.6	6.9	11.0	7.9
Thermodesulfobacteriota	*Desulfovibrio*	1.5	0.8	1.8	1.4	0.8	1.1	1.2	1.6	1.4	0.6
Deferribacterota	*Mucispirillum*	0.7	0.0	0.0	0.0	0.0	0.0	0.0	0.6	0.0	0.0

Mycoplasmatota	*Anaeroplasma*	2.0	0.8	2.2	0.7	0.8	1.6	3.2	1.1	0.9	0.9
Pseudomonadota	*Pseudomonas*	0.0	0.0	1.9	0.0	2.0	0.0	0.0	0.0	0.6	0.0
Streptophyta	*Incertae_sedis*	0.0	0.0	0.0	0.0	0.0	0.4	0.0	0.0	0.6	0.0

CG – control group, TG – test group.
